# Developing a dancer wellness program employing developmental evaluation

**DOI:** 10.3389/fpsyg.2014.00731

**Published:** 2014-07-10

**Authors:** Terry Clark, Arun Gupta, Chester H. Ho

**Affiliations:** ^1^Division of Physical Medicine and Rehabilitation, Department of Clinical Neurosciences, Faculty of Medicine, University of CalgaryCalgary, AB, Canada; ^2^Hotchkiss Brain Institute, University of CalgaryCalgary, AB, Canada

**Keywords:** performing artists, wellness programs, developmental evaluation, screening, health

## Abstract

Wellness programs are being increasingly employed with performing artists. Given their aim of reducing injuries, injury tracking is commonly employed as an outcome measure. Evaluating the development and process of a wellness program can also enhance its effectiveness. Developmental evaluation offers one methodological framework within which to conduct such investigations. This paper reports on a 2-year process involving feedback from professional ballet dancers, management and artistic staff, and healthcare providers at a ballet company in order to develop a dancer screening and wellness program. Following a consultation phase, an initial program composed of an expanded medical team and annual injury prevention screen was proposed. Alongside implementation with 30 professional ballet dancers, formal and informal feedback was sought from stakeholders and members across all levels of the ballet company to facilitate ongoing development, evaluation, and revision of the wellness program. The use of a process informed by developmental evaluation helped identify strengths and limitations within the screening process. The collective expertise of the assessors was used to modify the components and process of the screen to strive for ecological appropriateness. The process also fostered buy-in from all involved. Participant feedback helped refine the medical team available to the dancers and influenced the treatment and referral pathways via which dancers are able to access each member of the medical team. Furthermore, reflective discussions with artistic and management staff brought to light potential interactions between repertoire programming, fitness, and injury patterns. This prompted a reconsideration of how artists are trained and supported. Evaluation methods that focus on experiences and insight gained during program development stand to result in more efficient screening programs and health-promotion models and, ultimately, healthier performing artists.

## INTRODUCTION

Due to the physical and mental demands inherent in pursuing their art, performing artists are at a significant risk of sustaining performance-related injuries. Incidence rates for professional ballet dancers range from 0.62 to 4.4 injuries per 1000 h of exposure to dance activities when examined prospectively (Nilsson et al., 2001; Allen et al., 2012). Although no prospective injury incidence data for musicians exist, retrospective data indicate that 56% of musicians will sustain a music-related injury throughout their careers (Zaza, 1998; James, 2000; Spahn et al., 2004). This research also indicates that the types of mental and physical health problems that performing artists experience are varied and complex. Anxiety and perfectionism are common among musicians (Marchant-Haycox and Wilson, 1992) and have been acknowledged to influence the likelihood of physical as well as psychological injury. For example, they have been found to increase susceptibility to musicians’ dystonia (Jabusch and Altenmüller, 2004). Similarly, it has been suggested that stress hardiness can moderate musicians’ susceptibility to injury and performance anxiety (Salmon et al., 1995). The prevalence of body image problems and inappropriate eating attitudes and behaviors has been found to be significantly higher among ballet dancers than controls (Ravaldi et al., 2003). Set against the backdrop of a profession in which “sick-days” are rarely an option, performing artists do not always achieve full rehabilitation following their injuries as evidenced by studies demonstrating impaired proprioception following injury among dancers (Leanderson et al., 1996; Clark and Redding, 2012).

Often deeply entrenched in tradition, performing arts institutions are at the early stages of recognizing their role in supporting the health and wellbeing of their artists (Brandfonbrener, 2004). At the tertiary level, many music schools still consider their primary role as training skilled, competitive, and successful musicians (Perkins, 2011). As we appreciate the complexity of these environments, research interest is being increasingly devoted to understanding the cultures within which performing artists are trained and developed (Burt and Mills, 2007; Welch, 2007; Jørgensen, 2009; Lewton-Brain, 2012).

In order to address the prevalence of health problems among performing artists, a growing number of arts institutions are developing and offering wellness programs to their artists (Chesky et al., 2006; Manchester, 2007; Potter et al., 2008; Atkins, 2009). The goal of many wellness programs is to improve artists’ health; but how to achieve this goal and determine the most effective strategy to tackle this complex challenge remains uncertain. A number of methodological options are available, such as on-site healthcare and support, screening programs, and educational initiatives, for example. However, given the idiosyncrasy of each environment within which these programs are implemented in terms of the perceptions and expectations of the people involved (i.e., dancers, artistic staff, management, healthcare providers) and the resources (i.e., financial, time, staffing) that the organization may be able to devote to a potential wellness program, it is unlikely that there will be a single “optimal” wellness program that is appropriate for all settings. That said, it is not our intention to suggest that methodologies such as those informed by the Sequence of Prevention Model are not applicable to a wide range of performing arts organizations (van Mechelan, 1997). Rather, what we are trying to suggest is that the method by which injury prevention interventions can be implemented will vary and be dependent upon situational constraints particular to each organization which need to be considered.

In response to this situation, this paper will introduce and discuss an ongoing research project designed to develop, evaluate, and revise a dancer wellness program with a professional ballet company. To clarify, in the present situation the term “medical team” refers to physicians and healthcare practitioners including physiotherapists, chiropractors, psychologists, naturopathic doctors, massage therapists, and Pilates instructors.

### PERFORMING ARTIST WELLNESS PROGRAMS

Although not a new concept, multi-faceted wellness programs for dancers and musicians have received scant discussion in the literature to date. What literature exists typically focuses on educational initiatives at post-secondary institutions (Chesky et al., 2006; Redding and Quested, 2006; Manchester, 2007; Atkins, 2009, 2013). In general, this body of literature demonstrates that an increasing number of performing arts schools offer a wide range of health and wellbeing provisions for their students. However, the literature also reveals that many students do not feel able to make use of the full benefit of health and wellbeing provisions within their institutions (Atkins, 2009). This could be resulting, in part, from the lack of clearly defined policies and strategies for how best to cohesively utilize available on-site health and wellbeing services at many post-secondary institutions (Atkins, 2013). However, recent initiatives such as Dance UK’s *Healthy Dancer Program*, Conservatoires UK’s *Musical Impact Project*, and the Performing Arts Medicine Association and the National Association of Schools of Music’s *Health and Safety Accreditation Program* are working to develop united, national strategies to address this.

Injury screening programs are becoming increasingly employed within the performing arts as key components of wellness programs at both the student and professional levels (Liederbach, 1997). In addition to facilitating health promotion and injury prevention among a cohort (Fuller and Peirce, 2009), effective screening programs can provide a variety of benefits to artists and those in charge of supporting and ensuring their health (Clark et al., 2013). At the most basic level, screening programs are useful for identifying intrinsic risk factors that might predispose an individual to injury (Molnar and Esterson, 1997; Solomon, 1997). Screening programs can provide an assessment of the health status of a group, be that a professional ballet company or a year cohort within a higher education arts institution (Potter et al., 2008). Longitudinal screening programs can help establish norms for performance-related parameters (Fuller and Peirce, 2009), helping develop a clearer understanding of the physical and mental demands of training and performing (Williamon et al., 2009). Finally, screening programs can be useful from a programmatic perspective, depending upon how they are implemented, in that they can be used to assess the impact of training and performing programs on student or professional performing artists across time (Redding and Quested, 2006).

### EVALUATING WELLNESS PROGRAM DEVELOPMENT AND EFFICACY

Given that one of the main objectives of many wellness programs is a reduction of injuries, injury tracking is a common component (Ojofeitimi and Bronner, 2011; Allen et al., 2012, 2013; Liederbach et al., 2012; Ekegren et al., 2013). Assessing the development of the overall wellness program and the process of the components within it can also contribute to enhancing the effectiveness of a wellness program. Such assessment, together with the ensuing discussions, can also help foster buy-in and engagement to the wellness program from all involved. The collection of stakeholder (i.e., artist, healthcare provider, management, and artistic staff) feedback can be used to help inform the development of the overall wellness program, such as the content and process by which a screening program is implemented, its interaction with broader health-promotion activities, and the content and delivery of those broader health-promotion activities. Once considered, however, all components of a wellness program should be subjected to the necessary empirical evaluation to ensure validity and warrant continued use. As such, collecting stakeholder feedback can help increase the overall efficiency and efficacy of a wellness program.

Developmental evaluation offers one methodological framework through which to conduct such investigations. Developmental evaluation has been defined as “*evaluation processes and activities that support program, project, product, personnel and/or organizational development*” (Patton, 1994, p. 317). Developmental evaluation was initially developed for use with social innovations within complex environments and is underpinned by the complexity theory (Patton, 2011). In the light of this, Patton (2011, p. 7) notes that “*Developmental evaluation tracks and attempts to make sense of what emerges under conditions of complexity, documenting and interpreting the dynamics, interactions, and interdependencies that occur as innovations unfold.*” Developmental evaluation recognizes that identifying the solutions to complex problems, such as determining the best methods to support performing artists’ health and wellbeing, is not a linear process. Instead, the process involves jumping back and forth between problem and solution, continually revising the approach as new information is obtained (Gamble, 2008).

In discussing the “doing” of developmental evaluation, Gamble (2008) identifies three key features of a developmental evaluation within social innovation:

(1) Framing the issue: “*As innovators work on [an issue], their understanding moves from a vague understanding to increased clarity*” (Gamble, 2008, p. 20). Insight gained along the way may result in a shift in thinking, prompting another phase of uncertainty and clarification.
(2) Testing iterations: “*New ways of doing something are tried, often based on feedback loops and perspective about changing needs and demands, which can lead to improvements*” (Gamble, 2008, p. 20). Developmental evaluation helps identify and make clear the feedback loops and perspective that contribute to greater clarity and understanding.
(3) Tracking the trajectory of the innovation: “*Developmental evaluation records the roads not taken, unintended consequences, incremental adjustments, tensions and sudden opportunities*” (Gamble, 2008, p. 20). This insight can then be of value to others trying to solve similar problems, or in the application of the lessons learned to other scenarios.

Recognizing that developmental evaluation may not be an appropriate methodological choice within all contexts or for addressing all issues, it has been recommended that a number of characteristics exist within an organization in order for developmental evaluation to be effectively implemented (Gamble, 2008, p. 19):

• Innovation is identified as a core value.
• There is an iterative loop of option generation, testing, and selection.
• Board and staff are in agreement about innovation and willing to take risks.
• There is a high degree of uncertainty about the path forward.
• There are resources available for ongoing exploration; and
• The organization has a culture suited to exploration and enquiry.

Unlike formative or summative methods of evaluation that are useful to improve and evaluate the effectiveness of established models, developmental evaluation is useful for situations in which the end point, and/or methods of reaching the end point, is not well known or understood (for comparisons of developmental evaluation with traditional evaluation approaches, see Fagan et al., 2011; Patton, 2011). While many professional performing arts organizations and performing arts schools have a desire to support the health and wellbeing of their artists, the optimal methods for achieving this goal remain as yet unclear. Hence, a novel, innovative approach to understanding effective injury prevention and health promotion for student and professional performing artists is required.

### RESEARCH AIMS

The overarching aim of this project is to develop and evaluate a dancer wellness program within a professional ballet company. Features of developmental evaluation have been utilized to facilitate this process. Specifically, this paper reports on a unique, 2-year, developmental process involving feedback from professional ballet dancers, management, artistic staff, and healthcare providers at a ballet company in order to develop a comprehensive wellness program that includes on-site dancer screening. As well, the potential utility of developmental evaluation as a methodological approach to facilitate this process will be explored.

## MATERIALS AND METHODS

### RESEARCH SETTING

This initiative takes place within a North American full-time professional ballet company of 32 contracted dancers. The repertoire performed by the company is a mixture of classical ballet and contemporary works. For the 2013–2014 season, the company has a total of 46 contracted weeks and will give 61 performances, both at home and on tour throughout Canada. For the present discussion, a performance refers to either a full-length production or a series of smaller works presented within a single mixed-bill event whereby the mixed-bill refers to one performance. The dancers take part in 30 h of contracted dance activities (rehearsals and performances) each week.

In satisfying Gamble’s (2008) recommended organizational characteristics, embracing innovation, and risk taking are at the core of the ballet company. This is reflected in the company’s mission statement and is evidenced through their mounting of classical repertoire and the regular creation of new repertoire. While improving the health of their dancers is of fundamental concern to the company, there is a high degree of uncertainty over how best to achieve this. In working toward this initiative, key stakeholders across the company have expressed a willingness to engage in a collaborative, iterative process. Finally, three years of funding have been secured to facilitate the development and evaluation of this site-specific dancer wellness program.

This initiative stemmed from a joint interest between the ballet company and the University of Calgary to create an innovative, interdisciplinary wellness program for the ballet company’s dancers, with a clinical goal to address the often times underserved physical and psychosocial issues of the dancers, and a research goal to study the impact of the implementation of a wellness program within the ballet company. This led the ballet company to connect with the Division of Physical Medicine and Rehabilitation (PM&R) within the Department of Clinical Neurosciences. They approached the Division of PM&R because of their unique skill set that encompassed both specialty in rehabilitation and sports medicine. These shared interests and goals created the framework to facilitate the presently reported initiative. Furthermore, this collaboration was instrumental for securing the three years of funding mentioned above.

### RESEARCH DESIGN

The research reported in the present manuscript is longitudinal in design and is site-specific to this particular ballet company. Rather than testing the efficacy of a set model of health promotion (a dancer wellness program in this case), this research focuses on the continual development of the wellness program as new insight and knowledge emerges. This is in contrast to traditional formative and summative methods of evaluation. Instead, this research employs features of developmental evaluation (Patton, 2011) to guide the ongoing evaluation and development of a novel wellness program.

Referring to Gamble’s (2008), three key features of conducting a developmental evaluation, the following overarching questions have been formed to guide the development of this initiative:

(1) Framing the issue: What are the needs and problems to be addressed specifically within this ballet company?
(2) Testing iterations: What methods exist to allow us to address these needs and problems?
(3) Tracking the trajectory of the innovation: (a) What impact are our activities having on everyone involved in the program; and (b) What new opportunities and challenges are emerging as the program progresses and how might we be able to respond to them?

Specifically, the following activities have been undertaken over the past two years in order to develop, implement, evaluate, and revise the overall wellness program:

• Ten months of initial meetings were held with the management and artistic staff of the ballet company to ascertain wants and needs. Fundamentally, the ballet company was concerned about the level and frequency of injuries that their dancers were sustaining. Due to this, they were seeking specialist musculoskeletal care to aid in the treatment of and rehabilitation from those injuries. Additionally, they aimed to develop and implement a long-term health-promotion and injury prevention strategy to decrease the number of injuries occurring.
• From this, we proposed to develop and make available to the dancers an expanded medical team and conduct an annual injury prevention screen and injury surveillance.
• Prior to the implementation of any aspect of this program, ethical clearance was obtained from the relevant institutional human research ethics board. Each participant was provided with an information letter and signed an informed consent prior to commencing any further aspect of the initiative.
• In the 2012–2013 season, 22 of the company dancers took part in the first round of screening which utilized the Dance/USA Taskforce on Dancer Health Annual Post-Hire Health Screen protocol (Dance/USA Task Force on Dancer Health, 2013), conducted by eight assessors (physicians, allied health professionals, and one researcher). This screening protocol collects the following information: medical and orthopedic history, mental health, body composition, aerobic fitness, postural assessment, hypermobility, range of motion, strength, balance, and functional movement analysis. Prior to conducting the screen, each assessor was trained on how to complete the assessments they were responsible for using an instructional video created by the Dance/USA Taskforce on Dancer Health. Seven of the eight assessors who conducted the screen were healthcare practitioners who consulted to the ballet company and provided on- or off-site treatments for the dancers. The eighth assessor was a researcher. None of the assessors were employees of the ballet and all received payment for treatments either from the dancers themselves, from the dancers’ health insurance provider, or through billing the regional healthcare service.
• Following dissemination of results back to the dancers by the team physician and researcher, who were two of the eight screening assessors, all dancers and assessors were asked to complete an evaluation survey of the screening process. The dancers’ evaluation contained 10 short answer questions pertaining to perceived enjoyment, usefulness, applicability, and appropriateness of the components and process of the screen. The assessors’ evaluation contained 6 short answer questions addressing areas similar to those in the dancers’ form.
• The screening and survey results were next presented back to members of the management and artistic staff as well as dancer representatives by the researcher and team physician for discussion. These discussions, and the data upon which they were based, resulted in modifications to the screening protocol and the overall wellness program for the following season.
• Thirty of the company dancers took part in the second round of screening in the 2013–2014 season, again conducted by the same eight assessors. The 30 dancers comprised the 22 dancers from the first round of screening, six dancers who were new to the company, and two returning dancers who was not able to participate in the first round of screening due to illness.
• Following dissemination of results back to the dancers, again by the team physician and researcher, all assessors were again surveyed.
• Survey and screening results were presented back to members of the management, artistic staff, and dancer representatives by the researcher and team physician in order to continue developing the screening protocol and the overall dancer wellness program. Alongside these more formal evaluation activities, informal evaluation and feedback was sought from all parties involved in an ongoing process.

### QUALITATIVE DATA TREATMENT

As the goal of collecting formal and informal feedback was to establish contextualized perspectives of the thoughts and perceptions regarding the developing dancer wellness program from those involved, the analysis of the data obtained was data-driven rather than theory-driven (Charmaz, 2003). Specifically, content analysis involved identifying individual points of interest, or comments representing a single idea, within the written and verbal feedback obtained (Côté et al., 1993). These points of interest were labeled and grouped into categories with other similar points of interest. Finally, these categories were grouped together into general themes. Anonymized quotes representing these categories and general themes are presented below in section “Results.”

No identifiable screening or survey data were presented back to the management, artistic staff, or dancer representatives at any point. All data were blinded so as to ensure anonymity. The dancers did receive their own screening results back during private consultations, but these were the only occasions during which identifiable data were ever discussed.

## RESULTS

Through these formal and informal evaluation activities, a wide range of feedback and information was obtained. Thematically, this feedback was grouped into three overarching areas: (1) continued development of the screening protocol; (2) continued development of the wellness program; and (3) continued development of the ballet company’s approach to rehearsal. The results are presented below according to these three areas.

### CONTINUED DEVELOPMENT OF THE SCREENING PROTOCOL

Evaluation of the two rounds of screening identified strengths and benefits of the protocol, as well as challenges or limitations requiring further consideration.

#### Reported strengths and benefits

Dancers and assessors enjoyed taking part in the screen with assessors appreciating the collaborative nature of the process. Assessors also enjoyed the opportunity to evaluate the dancers’ movements in ways they would not normally be able to do:

“It gave me time to evaluate the dancers’ movements in a way that I would not otherwise check unless they were having a problem. It was a great chance to identify any health and musculoskeletal issues that a dancer may have before it leads to an injury (assessor).”

#### Reported challenges or limitations

Dancers and assessors both expressed concerns regarding the dissemination of the findings following the first round of screening. Dancers felt they did not receive sufficient feedback and were unclear about how to follow up on personal recommendations: “*The screening felt more for the medical team than for the dancers this first time around. I understand that has to happen in the beginning, I just look forward to when we get to hear more about the results and diagnoses (dancer).*” Also, assessors requested more of the results so that they would feel better equipped to work with the dancers in pre/re-habilitation scenarios: “*If we received more of the results of the tests we would be able to link deficiencies with injuries which would help us when creating pre/rehabilitation plans for the dancers (assessor).*”

Concerns were also raised regarding the location of the screening sessions. The first round of screening was conducted at the clinic of the company physician. This was chosen because all required equipment was at the clinic and it was felt that bringing all of the dancers to the clinic would provide them with a good opportunity to see the clinic, hence increasing comfort and familiarity with it. However, many of the dancers do not own a car and public transportation is limited which meant that getting to the clinic proved complicated.

Feedback obtained from the assessors following the second round of screening identified specific tests within the screening protocol that warrant further investigation to confirm their relevance and validity, as well as potentially useful aspects that were not being assessed in the protocol. The decision was made to follow this particular protocol for the first two rounds of screening so as to facilitate the aims behind its creation, namely the use of a standardized protocol that would contribute to a large pool of anonymous data that could contribute to knowledge of injury and illness and dance (Dance/USA Task Force on Dancer Health, 2013). However, going forward there is the desire to either modify or add to the protocol so as to ensure that it is appropriate for use with this particular company. Future research identifying balance assessments that are valid and reliable for use with dancers seems warranted. Static and dynamic balance assessments, such as the star excursion balance test, have shown potential utility for use with adolescents, however their validity for use with professional ballet dancers remains unknown as yet (Emery et al., 2005; Batson, 2010). Additionally, the dance Aerobic fitness test (Wyon et al., 2003) and subsequent high-intensity dance-specific fitness test (Redding et al., 2009) have been demonstrated to be capable of assessing cardiovascular fitness levels of dancers. Furthermore, given the prevalence of disordered eating among ballet dancers (Ravaldi et al., 2003, 2006; Thomas et al., 2005), research examining eating behaviors, body image, and psychological co-morbidities in dancers, together with an identification of validated assessment tools, could offer a significant contribution to the field.

#### Development of the protocol

Through reflection and discussion regarding these strengths and limitations, modifications were made to the protocol through the identification and trialing of new strategies and approaches and are discussed below. Some of these new strategies, such as modifications to the location and logistics of the screen, were implemented for the 2013–2014 screen while others, such as new assessment protocols within the screen, will be implemented in the 2014–2015 screen.

The second round of screening was held at the company’s studios as opposed to the clinic of the company physician, a change greatly appreciated by the dancers. The timings and logistics of the screening process were also changed which resulted in a quicker, smoother progression through the screen for the dancers.

The process by which the results and findings were provided to the dancers was changed for the second round of screening as well. In the first round, the dancers met with the company physician to debrief their results as a final station at the conclusion of their screen. During the second round, the dancers met with the company physician two weeks after their screen. This allowed the medical team time to compile all of the results from the screen and discuss and create summaries and recommendations for each dancer. This resulted in a more comprehensive conversation between each dancer and the company physician about their individual results and the potential implications of those results. Greater discussion of the results with the medical team following the second screen facilitated the development and delivery of pre-habilitation treatment plans for the dancers.

As discussed above in section “Reported Challenges and Limitations,” the identification of questionable tests and gaps within the screening protocol will form the basis of future investigations in order to improve the efficacy and ecological validity of the screening protocol. Such investigations will aim to identify the aspects most relevant for assessment (i.e., physiological, biomechanical, and psychological abilities and capacities) and the most appropriate methods for testing those areas.

### CONTINUED DEVELOPMENT OF WELLNESS PROGRAM

In addition to helping develop the screening protocol, the evaluation process has also assisted with the development of the overall wellness program. Acquiring a clearer understanding of the physical and mental health needs of the dancers has helped to identify the types of healthcare practitioners required to support those needs. For instance, 7 out of 21 dancers in the first round of screening scored positive on elements of the mental health portion of the screen. In the second round of screening, 7 out of 30 dancers expressed an interest in nutritional counseling and another 7 out of 30 dancers requested assistance with smoking cessation. This has resulted in an expanded medical team available to the dancers, both on-site and by referral. In particular, the medical team now includes members who are able to address mental health concerns, offer nutritional counseling, and provide assistance with smoking cessation.

As the medical team has expanded, a necessary issue to consider has been ensuring that the treatment and referral pathways by which the dancers are able to access the various members of the medical team are clarified and well communicated to the dancers. Not only is this information of value to the dancers, it also helps the medical team and the artistic and management staff understand how dancers move through the treatment and rehabilitation process.

Aside from the evaluation activities, the screening process itself identified a number of common areas of concern, or potential deficits, among the dancers. During the second round of screening, 11 and 15 out of 30 dancers scored positive for postural instability when balancing on their left and right ankles, respectively. Postural stability was assessed using a one-leg static balance task in which the dancer stands on one leg in parallel position with the opposite foot off the ground in a hip flexed/knee flexed position in which the raised leg does not make contact with the standing leg with their eyes closed. Further evaluation is recommended for any dancer who is not able to balance for at least 30 s (Dance/USA Task Force on Dancer Health, 2013). The dancers’ cardiovascular fitness was assessed using a 3-min step test (adapted from Golding et al., 1989). On a grading scale from 0 to 6 where 0 is “Excellent” and 6 is “Very Poor,” seven dancers scored 1 or lower and three dancers scored 3 or lower. For dancers scoring 3 or lower, supplemental aerobic training is recommended (Dance/USA Task Force on Dancer Health, 2013). In addition to these common areas of concern, individual points of concern were identified for many of the dancers. These included time-loss injuries in the 12 months prior to the screen as self-reported by 14 out of 30 dancers and 21 of the 30 dancers reporting experiencing ongoing musculoskeletal-related issues at the time of screening.

For many of these individual and common areas of concern, there is the possibility for them to be addressed on either an individual or a group basis, both of which present logistical pros and cons. While individual remedial work ensures specificity and detailed attention for each dancer, it is also financially prohibitive. Although more cost-effective, scheduling group supplemental training classes for all dancers becomes difficult when needing to work around busy rehearsal schedules. Group supplemental training classes also risk lacking the necessary specificity to adequately address each dancer’s particular area of concern. The screening process assists with identifying relevant areas of focus for supplemental training for the dancers; determining the most appropriate way to implement such classes, however, requires further exploration.

### CONTINUED DEVELOPMENT OF THE BALLET COMPANY’S APPROACH TO REHEARSAL

Over time, health surveillance via screening and injury tracking activities, together with ongoing evaluation and reflection, will allow for the development of a better understanding of some of the intrinsic and extrinsic risk factors faced by the dancers in this company. This in turn is facilitating a reconsideration of how dancers are trained and supported.

Reflective discussions with the artistic staff regarding the screening findings, such as the cardiovascular fitness results discussed above, have brought to light issues pertaining to dancers’ typical rehearsal processes. This has led to questioning potential interactions between repertoire programming, fitness, and injury patterns. The first performance of the 2012–2013 season was a mixed bill which saw many, if not all, of the company dancers rehearse and perform technically and physically demanding roles. The first performance of the 2013–2014 season, meanwhile, was a classical ballet which saw only a few dancers rehearsing and performing technically and physically demanding roles. Comparing the early parts of these two seasons, the artistic staff noted considerably poorer physical fitness levels in many of the dancers during the 2013–2014 season compared with the 2012–2013 season. Coincidentally, injury rates (as perceived by the artistic staff) appeared to be much higher during the 2013–2014 season compared with the 2012–2013 season. Although prospective injury tracking was not in place during the early part of the 2012–2013 season to confirm these perceived differences in injury patterns, this is still an interesting observation that concurs with the following research.

Research has noted that typical dance training and rehearsal do not inherently impact significantly upon dancers’ fitness and strength capacities (Baldari and Guidetti, 2001; Angioi et al., 2007). This is thought to be due to the intermittent, stop-and-start types of activities common within dance rehearsal that do not place significant stress on the aerobic system (Chatfield et al., 1992; Wyon et al., 2007; Twitchett et al., 2009, 2010a). As a result, classes and rehearsal are thought to place difference demands on the dancers’ cardio-respiratory system than performances (Wyon et al., 2004; Wyon and Redding, 2005). It is becoming increasingly clear that dancers, and performance quality, benefit from increased fitness and strength levels (Brown et al., 2007; Koutedakis et al., 2007). Furthermore, lower levels of fitness and strength have been linked to increased injury susceptibility and severity (Koutedakis et al., 1997; Twitchett et al., 2010b).

The observations from the ballet company’s artistic staff discussed above, when taken together with earlier research, raise potential implications for the programming of a ballet company’s season. Furthermore, it impacts how a company prepares and supports its dancers in terms of their fitness. The potential health and performance benefits obtainable through the employment of supplemental training programs and principles of periodization for dancers are being increasingly discussed (Koutedakis et al., 1999; Twitchett et al., 2010a, 2011; Wyon, 2010).

## DISCUSSION

The primary aim of the initiative reported in this paper is the ongoing development and evaluation of a site-specific dancer wellness program within a professional ballet company. This longitudinal process has been facilitated by developmental evaluation (Patton, 2011) and, more specifically, the three key features of a developmental evaluation as identified by Gamble (2008): (1) Framing the issue; (2) Testing iterations; and (3) Tracking the trajectory of the innovation. As such, this paper has also sought to explore the potential utility of developmental evaluation as a methodological approach to facilitate the above aim.

Following an initial consultation phase (Framing the issue), existing models of best practice were identified in the literature and subsequently trialed and evaluated (Testing iterations). Throughout this process, formal and informal feedback was sought from stakeholders and members across all levels of the ballet company via reflective discussions. The insight gained has facilitated the development and ongoing revision of the screening protocol, the wellness program, and the ballet company’s approach to rehearsal. The insight has subsequently impacted how the physical and mental health of the dancers is supported and prompted a reconsideration of how the dancers train throughout the season. All these have resulted in a reframing of the issue as a clearer understanding of the mental and physical health needs of this cohort are obtained. This clearer understanding of the dancers’ health needs, when considered within the context of this particular company, will facilitate greater clarity in terms of how best to address the emerging health needs (Tracking the trajectory of the innovation).

It is not the intention of this paper to suggest that more traditional forms of evaluation, such as formative and summative methodologies, are not appropriate for use in the development and evaluation of wellness programs for performing artists. Given that many wellness programs aspire to improve the health and wellbeing of performing artists, an indicator of changes to artists’ health will remain an essential component. Indeed, in the present scenario the ballet company management stated their primary objective in this initiative as supporting the health and wellbeing of their dancers. Further to this, they wanted to ensure as much as possible that their dancers would be adequately equipped to meet the requirements presented by the artistic staff and guest choreographers. To this end, prospective injury tracking was implemented at the ballet company at the beginning of the 2013–2014 season in order to better understand injuries and injury patterns within this cohort and any changes to those injuries and patterns. While the use of a reliable method of injury surveillance will be central to these aims (Liederbach et al., 2012), the employment of methods that can shed light upon *why* changes to artists’ health are occurring—or are not occurring—are also required. The use of a methodological approach informed by developmental evaluation can assist with obtaining this level of information.

Demonstrating the value of combining outcome- and process-focused methodologies, Redding and Quested (2006) discuss how reflection and discussion between dance scientists and teachers facilitated advancements in learning and teaching within a dance conservatoire. Following the collection of screening data at the beginning of the student’ academic year, discussions between research and teaching staff were held which resulted in the development and implementation of supplemental training programs in an effort to address some of the areas of concern identified within the screening program. The screening program was then used again at the conclusion of the students’ academic year to evaluate the effectiveness of the supplemental training programs for effecting change. Concluding, Redding and Quested (2006) point to the heightened collaboration between researchers and teaching staff and the personal empowerment of staff and students that resulted from the implementation of these combined methodologies. This conclusion concurs with the value of employing a mixed-method, iterative process such as that used in the present investigation.

### FUTURE RESEARCH

The developmental process employed in the present investigation has brought to light a number of areas that could benefit from further investigation. The assessor evaluation of the screening protocol questioned the validity of some of the tests employed and has identified other aspects that could benefit from being assessed. While sports science has produced a wealth of information that can be used to inform screening protocols for performing artists (Allen et al., 2013), research within the performing arts has highlighted how such methods require testing to ensure adequate ecological validity prior to implementation (Quin et al., 2008; Clark et al., 2010). Furthermore, as our understanding of the specific physical and mental capacities most relevant to performing artists increases, ensuring that screening protocols employ appropriate tests for assessing those capacities will be essential (Batson, 2010; Ruemper and Watkins, 2012).

Performing artists are susceptible to a range of psychosocial concerns in addition to physical complaints (Noh et al., 2003, 2005; Adam et al., 2004). While the screening protocol employed in the present investigation does include seven questions pertaining to mental health, further research in order to identify psychometrically valid tools capable of identifying potential psychosocial health concerns remains needed. The Athletic Coping Skills Inventory (ACSI-28) has been employed previously with Korean ballet dancers (Noh et al., 2005). However, the reliability and validity of the ACSI-28, or any other existing questionnaire, for use with professional ballet dancers has yet to be demonstrated.

As mentioned above, screening and injury tracking activities are useful for helping to identify common areas of concern or deficits in the capacities of a specific cohort that would benefit from remedial action. What screening and injury tracking are less capable of determining are the most appropriate methods for addressing these areas of concern or deficits. Limited resources of time and finances will always be issues for performing arts organizations. Furthermore, pre-existing perceptions of what should be included in a student or professional artist’s typical activities can either facilitate or impede the implementation of new activities (Redding and Quested, 2006). Knowledge translation activities that can assist in the implementation of evidence-based knowledge gained in the treatment room and research laboratory into the studio are sorely needed in the performing arts (Clark and Lisboa, 2013).

## CONCLUSION

When used in conjunction with a robust injury tracking protocol, collecting feedback from all parties involved in a wellness program for performing artist can provide a range of benefits. In the present study, the use of a process informed by developmental evaluation facilitated the ongoing development of a site-specific screening protocol, wellness program, and approach to supporting and training dancers. More specifically, participant feedback helped identify limitations or challenges within the screening process. The collective expertise of the assessors was used to modify the components and process of the screen to strive for ecological appropriateness. The process also fostered buy-in from all involved. Participant feedback helped refine the medical team available to the dancers and influenced the treatment and referral pathways via which dancers are able to access each member of the medical team. Furthermore, reflective discussions with artistic and management staff brought to light potential interactions between repertoire programming, fitness, and injury patterns. This prompted a reconsideration of how the artists are trained and supported. The present iterative process, involving a focus on the experiences and insight gained during the development of a wellness program, stands to result in contextually relevant, efficient screening programs and health-promotion models and, ultimately, healthier performing artists.

## Conflict of Interest Statement

The authors declare that the research was conducted in the absence of any commercial or financial relationships that could be construed as a potential conflict of interest.
